# Surgical outcomes of EA-MIDCAB vs. full sternotomy CABG in multi-vessel coronary artery disease: a retrospective cohort study

**DOI:** 10.1186/s13019-026-03891-7

**Published:** 2026-02-17

**Authors:** Hemn Abdulrahman Abdullah, Darya Nadir Saeed, Sherzad Ali Ismael, Marwan Abdullah Barzani, Blnd Azad Ismail, Marwan Tayyeb Tahir, Abdullah Haider Flayeh

**Affiliations:** 1 Cardiovascular Surgeon, Ministry of Health, Erbil, Iraq; 2 Professor of Community Medicine, Nutrition and Dietetics Department, Tishk International University, Erbil, Iraq; 3 Intern Doctor, Hawler Teaching Hospital, Erbil, Iraq; 4 College of Medicine, Hawler Medical University, Erbil, Kurdistan Region Iraq; 5 Department of biology, college of science, Salahaddin University-Erbil, Erbil, Kurdistan region Iraq

**Keywords:** EA-MIDCAB, Multivessel disease, Coronary artery bypass, Minimally invasive surgical procedures, Periareolar approach

## Abstract

**Background:**

Minimally invasive coronary artery bypass techniques are increasingly adopted to reduce surgical trauma and enhance recovery. This study aimed to compare early clinical outcomes of endoscopic-assisted minimally invasive direct coronary artery bypass (EA-MIDCAB)—predominantly via a periareolar incision—with conventional full sternotomy coronary artery bypass grafting (CABG) in patients with multivessel coronary artery disease.

**Methods:**

We retrospectively analyzed 393 patients diagnosed with multivessel coronary artery disease who underwent coronary artery bypass grafting (CABG) between February 2021 and May 2025. Of these, 332 underwent endoscopic-assisted minimally invasive direct CABG (EA-MIDCAB), and 61 underwent conventional full sternotomy CABG. Baseline characteristics, intraoperative and postoperative outcomes, and complication rates were compared between groups. Statistical analyses were performed using appropriate tests, with significance defined as *p* < 0.05.

**Results:**

Baseline characteristics and coronary stenosis distribution were comparable between groups. EA-MIDCAB patients demonstrated significantly lower cardiopulmonary bypass (CPB) usage (11.1% vs. 83.6%, *p* < 0.001), reduced red blood cell transfusion requirements (0.82 vs. 1.56 pints, *p* < 0.001), and fewer graft conduits (≥ 4 conduits: 0.6% vs. 19.2%, *p* < 0.001). Despite a higher prevalence of diabetes and hypertension, short-term outcomes—including mortality, renal dysfunction, and postoperative myocardial infarction—were similar between the two groups.

**Conclusions:**

EA-MIDCAB offers a safe and effective alternative to full sternotomy CABG for multivessel coronary artery disease, with reduced surgical trauma and transfusion needs.

## Background

Coronary artery disease (CAD) remains one of the leading causes of mortality worldwide, with multivessel involvement presenting significant risks and often requiring surgical intervention [1]. Coronary artery bypass grafting (CABG) is a well-established surgical technique for restoring myocardial perfusion in patients with severe CAD. Traditionally, this procedure has been performed through full sternotomy, offering broad surgical access but necessitating a large chest incision, which is associated with prolonged recovery and higher risk of complications [2, 3].

In recent years, minimally invasive direct coronary bypass (MIDCAB) has gained as an alternative approach, leveraging smaller incisions to potentially reduce recovery time, infection rates, and other postoperative complications [4, 5]. Despite these advantages, the adoption of Endoscopic-Assisted Minimally Invasive Direct Coronary Artery Bypass (EA-MIDCAB) remains limited, partly due to a lack of comprehensive comparative studies evaluating its efficacy relative to the full sternotomy approach, particularly in the context of multivessel CAD.

This study aims to fill this gap by comparing the outcomes of full sternotomy CABG and EA-MIDCAB via peri-areolar or submammary incision of a cohort of patients with multivessel CAD. By examining surgical and postoperative results, this research seeks to provide guidance to improve surgical decision-making and optimize patient outcomes for individuals with multivessel CAD.

## Methods

### Study design and setting

This retrospective cohort study was performed at Shar Hospital, a tertiary cardiac center in Erbil, Iraq, and involved patients who underwent either full sternotomy coronary artery bypass grafting or endoscopic-assisted minimally invasive direct coronary artery bypass through periareolar or submammary incisions from February 2021 to May 2025. A total of 393 patients satisfied the eligibility criteria, including 332 who received EA-MIDCAB and 61 who underwent full sternotomy CABG. Eligible patients exhibited angiographically verified multivessel coronary artery disease, characterized by significant stenosis, critical stenosis or total occlusion in three or more coronary arteries, irrespective of the exact vessels affected. Patients were excluded if they had one- or two-vessel disease, underwent concomitant procedures (e.g., valve surgery), or possessed incomplete perioperative data.

### Variable and outcome definitions

Coronary artery disease was defined angiographically as the presence of total occlusion, critical stenosis (> 90%), or significant stenosis (> 70%) in any major coronary artery, except in the left main coronary artery, where a threshold of > 50% stenosis was used. Triple-vessel disease was defined as involvement of the three main coronary arteries: the left anterior descending, left circumflex, and right coronary artery. Surgical duration was measured from the induction of general anesthesia to its completion. For intraoperative and postoperative laboratory parameters, the most clinically relevant value—either peak or nadir—was selected based on its clinical significance. Acute respiratory failure was diagnosed based on arterial blood gas analysis. Hypoxemic respiratory failure was defined as a partial pressure of oxygen (PaO₂) < 60 mmHg on room air, while hypercapnic respiratory failure was defined as a PaCO₂ > 50 mmHg with an accompanying pH < 7.35, also on room air. Postoperative myocardial infarction (Type 5 MI) was defined as a troponin elevation > 10 times the upper reference limit following CABG.

### Surgical technique

All surgical procedures were performed by a dedicated cardiac surgical team under the supervision of two cardiothoracic surgeons, following standardized protocols for both endoscopic-assisted minimally invasive direct coronary artery bypass (EA-MIDCAB) and full sternotomy coronary artery bypass grafting (CABG). In the EA-MIDCAB group, a limited anterior thoracotomy was typically performed via a periareolar incision in male patients and in some female patients, or through a submammary incision in female patients, at the level of the fourth or fifth intercostal space. This was usually accompanied by a secondary port at the third intercostal space to facilitate endoscopic instrumentation and visualization. Conduits such as the left internal thoracic artery (LITA), and radial artery grafts were harvested and utilized as needed. Graft configurations included sequential and loop grafts, tailored to the patient’s coronary anatomy and target vessels. In the full sternotomy CABG group, a median sternotomy was performed with wide pericardial exposure, allowing the use of multiple conduits including the LITA, radial artery, and saphenous vein grafts. Graft selection and configuration—whether single, sequential, or composite. Proximal anastomoses were constructed using side-clamping or full cross-clamping, depending on the case. Both surgical approaches allowed for off-pump or on-pump techniques as clinically indicated, depending on hemodynamic stability and target vessel accessibility. Following grafting, all patients were transferred to the intensive care unit (ICU) in stable condition, with inotropic support initiated as needed (e.g., dobutamine infusion at 3 ml/hr).

### Statistical analysis

All data were analyzed using Statistical Package of Social Sciences (SPSS) software for Windows, Version 26.0 (IBM Corp., Armonk, NY, USA). Continuous variables were assessed for normality using the Shapiro–Wilk test. As most variables demonstrated a non-normal distribution they were expressed as mean ± standard deviation (SD) with 95% confidence intervals (CI) and compared between groups using the Mann–Whitney U test. Categorical variables were summarized as frequencies and percentages and compared between groups using the Chi-square test, where > 20% of the expected cell counts were less than 5, Fisher’s exact test was applied. For contingency tables larger than 2 × 2, the Likelihood Ratio Chi-square was reported, cases with missing values were handled by pairwise deletion and were excluded only from the specific analysis in which the variable was missing. A two-tailed p-value < 0.05 was considered statistically significant.

## Results

A total of 393 patients were included in the final analysis, comprising 332 who underwent endoscopic-assisted minimally invasive direct coronary artery bypass (EA-MIDCAB) via periareolar or submammary incision and 61 who underwent full sternotomy coronary artery bypass grafting (CABG). Table 1 summarizes the baseline demographic, clinical, and laboratory characteristics of the two groups. Baseline characteristics were generally comparable between groups. However, diabetes mellitus was significantly more prevalent in the EA-MIDCAB group than in the full sternotomy group (69.3% vs. 54.1%, *p* = 0.021), as was hypertension (43.4% vs. 26.2%, *p* = 0.012). Additionally, HbA1c levels were higher in the EA-MIDCAB group (7.16 ± 1.56% vs. 6.56 ± 1.34%, *p* = 0.006).

**Table 1:** Baseline demographic and clinical characteristics of patients undergoing EA-MIDCAB and full sternotomy CABG group

Variables	EA-MIDCAB (*N* = 332)	Full Sternotomy CABG (*N* = 61)	*P*-value
Age (years)	62.0 ± 8.4 (61.1–62.9)	62.4 ± 9.0 (60.1–64.7)	0.787ᵃ
Gender (Male)	276 (83.1%)	51 (83.6%)	0.927ᵇ
BMI (Kg/m^2^)	29.4 ± 5.7 (28.8–30.0)	28.3 ± 3.9 (27.3–29.3)	0.239ᵃ
Diabetes Mellitus	230 (69.3%)	33 (54.1%)	0.021ᵇ*
Hypertension	144 (43.4%)	16 (26.2%)	0.012ᵇ*
Chronic Kidney Disease	34 (10.2%)	3 (4.9%)	0.191ᵇ
Heart Failure	30 (9.0%)	4 (6.6%)	0.527ᵇ
Triple vessel disease	257 (77.4%)	48 (78.7%)	0.826ᵇ
Ejection fraction (%)	53.3 ± 10.4 (52.1–54.4)	52.9 ± 11.0 (50.0–55.8)	0.811ᵃ
S. Creatinine (mg/dL)	1.22 ± 1.87 (1.01–1.42)	1.01 ± 0.22 (0.95–1.07)	0.717ᵃ
Hemoglobin (g/dL)	13.6 ± 5.3 (13.0–14.2)	13.4 ± 1.7 (12.9–13.8)	0.334ᵃ
WBC (x10^3^/ µL)	7.7 ± 1.9 (7.5–8.0)	8.9 ± 5.6 (7.4–10.0)	0.225ᵃ
Platelets (×10³/µL)	234 ± 72 (227–242)	238 ± 72 (219–257)	0.923ᵃ
HbA1c (%)	7.16 ± 1.56 (6.99–7.33)	6.56 ± 1.34 (6.19–6.93)	0.006ᵃ*

Values are presented as mean ± standard deviation (95% confidence interval) or n (%). BMI: body mass index; EF: ejection fraction; WBC: white blood cell count; S. Creatinine: serum creatinine. ᵃ Mann-Whitney U test, ᵇ Pearson Chi-square test. ᶜ Fisher’s exact test. *Statistically significant at *p* < 0.05

Table 2 presents the distribution of coronary artery stenosis—including total occlusion (TO), critical stenosis (CS), and significant stenosis (SS)—across major coronary arteries and branches in the EA-MIDCAB and full sternotomy CABG groups. The extent and severity of coronary lesions were generally comparable between the two groups. For instance, critical stenosis of the left anterior descending artery (LAD) was found in 78.9% of EA-MIDCAB cases and 75.4% of full sternotomy cases (*p* = 0.711), while total occlusion of the right coronary artery (RCA) occurred in 21.7% and 23.0% of cases, respectively (*p* = 0.488). No statistically significant differences were detected across the LAD, LCX, RCA, or their branches.

**Table 2:** Distribution of preoperative coronary artery stenosis among patients undergoing EA-MIDCAB vs. Full sternotomy CABG group

Coronary Artery and branches	EA-MIDCAB (*N* = 332)	Full Sternotomy CABG (*N* = 61)	*p*-value
Left main Coronary Artery	TO: 7 (2.1%) CS: 106 (31.9%) SS: 34 (10.2%)	TO: 0 (0.0%) CS: 22 (36.1%) SS: 8 (13.1%)	0.311ᵇ
Left Anterior Descending	TO: 38 (11.4%) CS: 262 (78.9%) SS: 23 (6.9%)	TO: 7 (11.5%) CS: 46 (75.4%) SS: 5 (8.2%)	0.711ᵇ
Left Circumflex Artery	TO: 29 (8.7%) CS: 234 (70.5%) SS: 28 (8.4%)	TO: 3 (4.9%) CS: 43 (70.5%) SS: 6 (9.8%)	0.822ᵇ
Right Coronary Artery	TO: 72 (21.7%) CS: 190 (57.2%) SS: 32 (9.6%)	TO: 14 (23.0%) CS: 41 (67.2%) SS: 3 (4.9%)	0.488ᵇ
Intermediate Branch	TO: 2 (0.6%) CS: 17 (5.1%) SS: 4 (1.2%)	TO: 0 (0.0%) CS: 0 (0.0%) SS: 3 (4.9%)	0.057^d^
First Diagonal Branch	TO: 2 (0.6%) CS: 67 (20.2%) SS: 11 (3.3%)	TO: 0 (0.0%) CS: 14 (23.0%) SS: 6 (9.8%)	0.105^d^
Obtuse Marginal 1	TO: 5 (1.5%) CS: 134 (40.4%) SS: 20 (6.0%)	TO: 1 (1.6%) CS: 30 (49.2%) SS: 0 (0.0%)	0.298^d^
Obtuse Marginal 2	TO: 3 (0.9%) CS: 29 (8.7%) SS: 3 (0.9%)	TO: 0 (0.0%) CS: 6 (9.8%) SS: 2 (3.3%)	0.400^d^
Posterior Descending Artery	TO: 3 (0.9%) CS: 97 (29.2%) SS: 15 (4.5%)	TO: 0 (0.0%) CS: 23 (37.7%) SS: 2 (3.3%)	0.095^d^

Values are presented as n (%). TO, Total Occlusion; CS, Critical Stenosis; SS, Significant Stenosis. ᵇ Pearson Chi-square, ^d^ Likelihood ratio Chi-square.

Table 3 summarizes key intraoperative outcomes. Surgery duration was slightly longer in the EA-MIDCAB group (4.80 ± 1.44 h) compared to the full sternotomy group (4.31 ± 1.10 h), with a statistically significant difference (*p* = 0.032). Cardiopulmonary bypass (CPB) usage differed significantly (*p* < 0.001); the majority of EA-MIDCAB cases were performed off-pump (88.9%), whereas most full sternotomy CABG cases required CPB (83.6%). Although urgency levels did not differ significantly (*p* = 0.065), a higher proportion of urgent and emergent cases occurred in the full sternotomy group. Additionally, the number of graft conduits used was significantly higher in the full sternotomy group (*p* < 0.001), with 19.2% of patients receiving 4 or more conduits compared to just 0.6% in the EA-MIDCAB group. Intraoperative hemoglobin was significantly higher in EA-MIDCAB patients (12.6 vs. 10.5 g/dL, *p* < 0.001).

**Table 3:** Intraoperative outcomes in patients undergoing EA-MIDCAB and full sternotomy CABG group

Variables	EA-MIDCAB (*N* = 332)	Full Sternotomy CABG (*N* = 61)	*P*-value
Surgery duration (hours)	4.80 ± 1.44 (4.63–4.96)	4.31 ± 1.10 (3.99–4.64)	0.032ᵃ*
Cardiopulmonary bypass			< 0.001^b^*
Off-pump	295 (88.9%)	10 (16.4%)	
On-pump	37 (11.1%)	51 (83.6%)	
Urgency of operation			0.065^d^
Elective	299 (90.1%)	50 (82.0%)	
Urgent	29 (8.7%)	11 (18.0%)	
Emergent	4 (1.2%)	0 (0.0%)	
No. of conduits			< 0.001^d^*
1	7 (2.3%)	0 (0.0%)	
2	211 (70.8%)	31 (54.4%)	
3	78 (26.2%)	15 (26.3%)	
4	1 (0.3%)	8 (14.0%)	
5	1 (0.3%)	3 (5.2%)	
Hemoglobin (g/dL)	12.6 ± 8.5 (11.7–13.6)	10.5 ± 2.3 (9.9–11.1)	< 0.001^a^*

Values are presented as mean ± standard deviation (95% confidence interval) or n (%). ᵃ Mann–Whitney U test; ᵇ Pearson Chi-square test; ^d^ Likelihood ratio Chi-square test. *Statistically significant at *p* < 0.05.

Postoperative recovery metrics are shown in Table 4. ICU stay duration and overall hospitalization were comparable between groups (*p* = 0.060 and *p* = 0.812, respectively). Hemoglobin, serum creatinine, and WBC counts showed no significant differences. However, platelet counts were significantly higher in the EA-MIDCAB group (187 ± 61 × 10³/µL vs. 154 ± 47 × 10³/µL, *p* < 0.001). Blood product usage was also significantly lower in the EA-MIDCAB group, including packed red blood cells (0.82 vs. 1.56 pints, *p* < 0.001), platelets (0.27 vs. 0.84 pints, *p* < 0.001), while plasma use and chest tube drainage were not significantly different.

**Table 4:** Postoperative outcomes in patients undergoing EA-MIDCAB and full sternotomy CABG group

Variables	EA-MIDCAB (*N* = 328)	Full Sternotomy CABG (*N* = 60)	*P*-value
ICU stay (hours)	18.7 ± 20.2 (16.5–20.9)	18.6 ± 10.5 (15.9–21.4)	0.060^a^
Hospitalization (days)	5.27 ± 2.05 (5.04–5.50)	5.12 ± 1.35 (4.77–5.47)	0.812^a^
S. Creatinine (mg/dL)	1.26 ± 0.94 (1.16–1.37)	1.19 ± 0.47 (1.06–1.32)	0.320^a^
White blood cells (x10^3^/ µL)	15.1 ± 4.8 (14.5–15.6)	17.0 ± 7.2 (14.9–19.0)	0.197^a^
Hemoglobin (g/dL)	9.88 ± 1.65 (9.69–10.07)	9.89 ± 1.44 (9.47–10.29)	0.786^a^
platelet (×10³/µL)	187 ± 61 (180–194)	155 ± 47 (141–168)	< 0.00^a^*
Packed red blood cell (pints)	0.82 ± 1.44 (0.65–0.99)	1.56 ± 1.64 (1.14–1.98)	< 0.001^a^*
Platelet (pints)	0.42 ± 1.30 (0.26–0.57)	1.07 ± 2.11 (0.52–1.61)	< 0.001^a^*
Plasma (pints)	0.95 ± 1.48 (0.77–1.12)	1.12 ± 1.61 (0.70–1.53)	0.423^a^
Chest tube drainage (mL)	962 ± 663 (890–1033)	1110 ± 881 (884–1336)	0.216^a^

Values are presented as mean ± standard deviation (95% confidence interval) or n (%).ICU, intensive care unit; S. Creatinine, serum creatinine. ᵃ Mann–Whitney U test. *Statistically significant at *p* < 0.05.

Postoperative complications were comparable between groups. Intraoperative and ICU mortality were low and not significantly different. Rates of respiratory failure, acute kidney injury, and ICU readmission were similar. Postoperative MI and stroke were rare in both cohorts. No statistically significant differences were observed in any complication category as shown in Table 5.

**Table 5:** Postoperative complications in EA-MIDCAB vs. Full sternotomy CABG group

Complications	EA-MIDCAB (*N* = 332)	Full sternotomy CABG (*N* = 61)	*P*-value
Intraoperative mortality	4 (1.2%)	1 (1.6%)	0.572ᶜ
ICU mortality	17 (5.2%)	0 (0.0%)	0.087ᶜ
Acute Respiratory failure	75 (22.6%)	15 (24.6%)	0.733ᵇ
Acute kidney injury	55 (16.6%)	8 (13.1%)	0.499ᵇ
Readmission to ICU	14 (4.2%)	0 (0.0%)	0.140ᶜ
Postoperative MI	9 (2.7%)	1 (1.6%)	-
Stroke	7 (2.1%)	1 (1.6%)	-

Values are presented as n (%), ICU, Intensive Care Unit; MI, Myocardial infarction. ᵇ Pearson Chi-square test. ᶜ Fisher’s Exact test.

## Discussion

This study aimed to compare the early surgical outcomes of endoscopic-assisted minimally invasive direct coronary artery bypass (EA-MIDCAB) with conventional full sternotomy CABG in patients with multivessel coronary artery disease. Our primary focus was to determine whether a less invasive approach could yield comparable or improved short-term clinical outcomes, without compromising safety. Notable findings included significantly lower cardiopulmonary bypass (CPB) use, less number of graft conduits, and lower blood product consumption in the EA-MIDCAB group. Despite these advantages, early postoperative complications, ICU mortality, renal dysfunction, and myocardial infarction rates were not significantly different between the two cohorts, supporting the non-inferiority of EA-MIDCAB.

study have shown that comaprision of multivessel between mics cabg and median sternotomy showing safer and effective in mics cabg. and do not necessarily demonstrate clinical superiority in major outcomes [6–8].

The observed difference in the number of graft conduits—significantly fewer in the EA-MIDCAB group—likely reflects tailored surgical strategies aimed at balancing effective revascularization with reduced invasiveness. Techniques such as sequential grafting and loop grafting are frequently employed in minimally invasive approaches to achieve complete coronary revascularization with fewer individual conduits. These configurations allow multiple coronary targets to be supplied from a single conduit, thereby reducing surgical trauma, graft manipulation, and operative time while maintaining perfusion adequacy [9]. Additionally, the significantly reduced need for blood product transfusions in the EA-MIDCAB cohort suggests decreased intraoperative blood loss and surgical trauma—hallmarks of minimally invasive techniques. This aligns with existing literature indicating that minimally invasive CABG approaches, such as EA-MIDCAB, are associated with reduced transfusion requirements due to smaller incisions, limited tissue dissection, and avoidance of full sternotomy [8, 10].

A notable shift in institutional surgical strategy occurred during the study period, with increasing preference for avoiding CPB in favor of off-pump EA-MIDCAB. This shift reflects broader trends in cardiac surgery aimed at reducing CPB-associated risks, such as reduced rates of blood-product transfusion and organ dysfunction, particularly in patients with comorbidities [11]. These benefits support the ongoing trend toward less invasive, pump-avoiding surgical strategies in cardiac revascularization.

The ability to achieve complete revascularization using a minimally invasive approach—while reducing intraoperative hemoglobin loss and transfusion needs—makes EA-MIDCAB particularly valuable in healthcare systems aiming to optimize postoperative resource utilization and improve patient satisfaction. Additionally, although the procedure carries a defined learning curve, outcomes improve significantly with increasing surgeon experience, A study of 300 robotic EA-MIDCAB cases found that complication rates declined quickly after just 50 cases. Most early complications were clustered in the first third of cases, indicating a short but steep learning curve [12], supporting the broader adoption of periareolar EA-MIDCAB in modern cardiac surgery programs.

This study has several important limitations. First, its retrospective and single-center design inherently introduces selection bias and limits the generalizability of the findings. Second, there was a notable imbalance in sample size between the groups, as most full sternotomy CABG procedures were performed during the earlier phase of our institutional practice. Third, intraoperative documentation for the full sternotomy group was frequently incomplete, which limited our ability to perform detailed comparisons regarding graft configurations, target sites of distal anastomoses, and rates of conversion to cardiopulmonary bypass. Finally, the absence of long-term follow-up data prevents assessment of the durability of revascularization and the incidence of late complications.

Future studies should involve prospective cohort designs, potentially using propensity score matching to balance baseline characteristics and procedural differences. Comparative analyses of various minimally invasive CABG approaches—such as periareolar, minithoracotomy, or anterolateral thoracotomy—will be essential to evaluate not only safety and efficacy but also patient-reported outcomes such as satisfaction and quality of life. As cosmetic considerations increasingly influence patient preferences, the question remains: can we make coronary artery bypass surgery both safer and more acceptable through smaller, aesthetic incisions without compromising clinical outcome?

## Conclusions

EA-MIDCAB demonstrated comparable safety and early clinical outcomes to full sternotomy CABG in patients with multivessel coronary artery disease, while offering advantages such as reduced CPB use, fewer graft conduits, and lower transfusion requirements. These findings support the feasibility of a minimally invasive, periareolar approach for multivessel CABG and highlight the need for further prospective studies to validate long-term outcomes and patient-centered benefits.

## Data Availability

No datasets were generated or analysed during the current study.

## Abbreviations

CABG: Coronary Artery Bypass Grafting
EA-MIDCAB: Endoscopic-Assisted Minimally Invasive Direct Coronary Artery Bypass
CAD: Coronary Artery Disease
CPB: Cardiopulmonary Bypass
ICU: Intensive Care Unit

## References

[1] Mensah GA, Fuster V, Murray CJL, Roth GA, Mensah GA, Abate YH, et al. Global burden of cardiovascular diseases and Risks, 1990–2022. J Am Coll Cardiol. 2023;82(25):2350–473. 38092509 10.1016/j.jacc.2023.11.007 PMC7615984

[2] Sandha DPA, Sandy Tjang Y, Faranita T, Karina K. Comparison of clinical outcomes after coronary artery bypass grafting (CABG) with percutaneous coronary intervention (PCI) in coronary artery disease (CAD) patients with kidney disorders: systematic review. Jurnal Health Sains. 2024;5(2):52–72.

[3] Besola L, Colli A, De Caterina R. Coronary bypass surgery for multivessel disease after percutaneous coronary intervention in acute coronary syndromes: why, for whom, how early? Eur Heart J. 2024;45(34):3124–31. 39056269 10.1093/eurheartj/ehae413

[4] Reuthebuch O, Stein A, Koechlin L, Gahl B, Berdajs D, Santer D, et al. Five-Year survival of patients treated with minimally invasive direct coronary artery bypass (MIDCAB) compared with the general Swiss population. Thorac Cardiovasc Surg. 2024;72(06):404–12. 37044119 10.1055/s-0043-1768035 PMC11379533

[5] Alaj E, Seidiramool V, Ciobanu V, Bakhtiary F, Monsefi N. Short-Term clinical results of minimally invasive direct coronary artery bypass (MIDCAB) procedure. J Clin Med. 2024;13(11):3124. 38892835 10.3390/jcm13113124 PMC11173179

[6] Ong ZX, Wu D, Sule JA, Chang G, Sazzad F, Luo H et al. Minimally invasive coronary artery bypass grafting in a Low-Risk Asian cohort: A Propensity-Score matched study. Braz J Cardiovasc Surg. 2024;39(4), e20220421. 10.21470/1678-9741-2022-0421 PMC11259139 39037968

[7] Raja SG, Garg S, Rochon M, Daley S, De Robertis F, Bahrami T. Short-term clinical outcomes and long-term survival of minimally invasive direct coronary artery bypass grafting. Ann Cardiothorac Surg. 2018;7(5):621–7. 30505746 10.21037/acs.2018.06.14 PMC6219940

[8] Huang G, Zhang H, Chi L, You B, Bo P, Sun G. Comparing perioperative outcomes following off-pump multi-vessel minimally invasive via a single left intercostal space incision with median sternotomy coronary artery bypass grafting: A single-center retrospective cohort study. Perfusion. 2023 Sep 2:2676591231194454. 10.1177/02676591231194454 37658740

[9] Rodriguez ML, Lapierre HR, Sohmer B, Glineur D, Ruel M. Mid-Term Follow-Up of minimally invasive multivessel coronary artery bypass grafting. Innovations: Technol Techniques Cardiothorac Vascular Surg. 2017;12(2):116–20. 10.1097/IMI.0000000000000353 28328569

[10] Teman NR, Hawkins RB, Charles EJ, Mehaffey JH, Speir AM, Quader MA, et al. Minimally invasive vs open coronary surgery: A Multi-Institutional analysis of cost and outcomes. Ann Thorac Surg. 2021;111(5):1478–84. 32961136 10.1016/j.athoracsur.2020.06.136

[11] Lamy A, Devereaux PJ, Prabhakaran D, Taggart DP, Hu S, Paolasso E, et al. Off-Pump or On-Pump Coronary-Artery bypass grafting at 30 days. N Engl J Med. 2012;366(16):1489–97. 22449296 10.1056/NEJMoa1200388

[12] Van den Eynde J, Vaesen Bentein H, Decaluwé T, De Praetere H, Wertan MC, Sutter FP, et al. Safe implementation of robotic-assisted minimally invasive direct coronary artery bypass: application of learning curves and cumulative sum analysis. J Thorac Dis. 2021;13(7):4260–70. 34422354 10.21037/jtd-21-775 PMC8339757

